# Effectiveness of Three Sampling Approaches for Optimizing Mapping and Preventive Chemotherapy against *Schistosoma mansoni* in the Western Part of Côte d’Ivoire

**DOI:** 10.3390/tropicalmed9070159

**Published:** 2024-07-14

**Authors:** Jean-Baptiste K. Sékré, Mamadou Ouattara, Nana R. Diakité, Fidèle K. Bassa, Rufin K. Assaré, Jules N. Kouadio, Gaoussou Coulibaly, Agodio Loukouri, Mathieu N. Orsot, Jürg Utzinger, Eliézer K. N’Goran

**Affiliations:** 1 Unité de Formation et de Recherche Biosciences, Université Félix Houphouët-Boigny, Abidjan 22 BP 582, Côte d’Ivoire; mamadou_ouatt@yahoo.fr (M.O.); diaknarose@yahoo.fr (N.R.D.); fidelebassa@ymail.com (F.K.B.); hrufinass@yahoo.fr (R.K.A.); jules.kouadio@csrs.ci (J.N.K.); gaoussoubrava@yahoo.fr (G.C.); lkagodio@yahoo.fr (A.L.); mathorsot@gmail.com (M.N.O.); eliezerngoran@yahoo.fr (E.K.N.); 2 Centre Suisse de Recherches Scientifiques en Côte d’Ivoire, Abidjan 01 BP 1303, Côte d’Ivoire; 3 Swiss Tropical and Public Health Institute, Kreuzstrasse 2, CH-4123 Allschwil, Switzerland; juerg.utzinger@swisstph.ch; 4 University of Basel, Petersplatz 1, CH-4003 Basel, Switzerland

**Keywords:** Côte d’Ivoire, mapping, preventive chemotherapy, sampling approach, *Schistosoma mansoni*, schistosomiasis, school-aged children

## Abstract

The elimination of schistosomiasis as a public health problem by 2030 is one of the main goals put forth in the World Health Organization’s roadmap for neglected tropical diseases. This study aimed to compare different sampling approaches to guide mapping and preventive chemotherapy. A cross-sectional parasitological survey was conducted from August to September 2022 in the health districts of Biankouma, Ouaninou, and Touba in the western part of Côte d’Ivoire. The prevalence and intensity of *Schistosoma mansoni* infection were assessed in children aged 5–14 years using three sampling approaches. The first approach involved a random selection of 50% of the villages in the health districts. The second approach involved a random selection of half of the villages selected in approach 1, thus constituting 25% of the villages in the health district. The third approach consisted of randomly selecting 15 villages from villages selected by approach 2 in each health district. The overall prevalence of *S. mansoni* was 23.5% (95% confidence interval (CI): 19.9–27.6%), 21.6% (95% CI: 17.1–26.8%), and 18.3% (95% CI: 11.9–27.1%) with the first, second, and third approach, respectively. The respective geometric mean *S. mansoni* infection intensity was 117.9 eggs per gram of stool (EPG) (95% CI: 109.3–127.3 EPG), 104.6 EPG (95% CI: 93.8–116.6 EPG), and 94.6 EPG (95% CI 79.5–112.7 EPG). We conclude that, although randomly sampling up to 50% of villages in a health district provides more precise population-based prevalence and intensity measures of *S. mansoni*, randomly selecting only 15 villages in a district characterized by low heterogeneity provides reasonable estimates and is less costly.

## 1. Introduction

Schistosomiasis is a water-based parasitic disease with trematodes of the genus *Schistosoma* known as the causative agent [1,2]. Classified as a neglected tropical disease (NTD) by the World Health Organization (WHO), schistosomiasis affects more than 236 million people worldwide, with an estimated global burden of 1.4 million disability-adjusted life years (DALYs). Most infections occur in sub-Saharan Africa [3].

In 2012, WHO published the NTD roadmap that set targets for 2020, emphasizing preventive chemotherapy as the main control strategy [4,5]. In the same year, partners and stakeholders endorsed the London Declaration on NTDs and expressed their commitment to support the WHO roadmap and its 2020 targets. This resolution enhanced political will and encouraged many countries to establish national schistosomiasis action plans and control programmes [6]. According to WHO guidelines, preventive chemotherapy is based on the prevalence of *Schistosoma* infection in school-aged children [7]. This implies prior knowledge about the distribution and extent of schistosomiasis endemicity to serve as a compass for preventive chemotherapy. In the absence of prior or up-to-date schistosomiasis data, mapping is particularly salient to guide the frequency of interventions and to delineate the area and target population for intervention [8,9]. As part of national schistosomiasis control programmes, WHO recommends school-based, district-wide mapping in endemic countries [10]. According to new WHO guidelines, released in 2022, it is recommended to evaluate the impact of preventive chemotherapy interventions after 3 to 5 rounds of mass drug administration, to help inform decisions from these programmes [11].

In Côte d’Ivoire, both *Schistosoma mansoni* (causing intestinal schistosomiasis) and *S. haematobium* (causing urogenital schistosomiasis) are endemic, though the distribution is focal [12]. Previous studies have indicated a predominance of *S. mansoni* in the western part of Côte d’Ivoire, with prevalence among school-aged children exceeding 80% in some villages [13,14]. *S. haematobium* mainly occurs in the central and northern parts of Côte d’Ivoire, while it is rarely found in the western part of the country [14,15]. The first national mapping of schistosomiasis was carried out in 2013 and 2014, using health districts as the unit of mapping to guide preventive chemotherapy [16]. The design of this initial mapping focused on school-aged children and randomly selected 15 schools per health district [16]. Monitoring was performed in sentinel sites from 2014 to 2022 and showed, as in many other countries, that several rounds of mass drug administration considerably reduced the prevalence and intensity of schistosomiasis, and hence, elimination as a public health problem might be conceivable in specific contexts [10,17]. However, measuring the impact of preventive chemotherapy is challenging, as data obtained from the initial mapping approach might not reflect the precise situation of the entire health district. In the opinion of WHO technical experts and supported by previous research, the proposed and widely used mapping approach with only 15 villages per health district might explain these observations [18,19]. Hence, there is a need to examine alternative mapping approaches and compare them with the current standard WHO approach.

This study aimed to compare the performance of the national standard sampling approach (i.e., a fixed number of 15 localities per health district) with more extensive sampling by randomly selecting up to 50% of localities within a health district. The results of this study might guide mapping and preventive chemotherapy and inform impact assessment of schistosomiasis in Côte d’Ivoire and elsewhere.

## 2. Materials and Methods

### 2.1. Ethics Statement

The study obtained ethical approval from the “Comité National d’Ethique des Sciences de la Vie et de la Santé” (CNESVS) of Côte d’Ivoire (reference no. 059-22/MSHPCMU/CNESVS-kp; issued on 10 May 2022). A few days before sample collection, the heads of household and all participants received detailed information on the objectives, field and laboratory procedures, and potential risks and benefits of the study. Written informed consent was obtained from the children’s parents or legal guardians. Data were kept confidential by using individual unique codes. Participation was voluntary, and children could withdraw at any time without further obligations. After the survey, all children aged 5–14 years in the whole study area were treated with praziquantel (the standard treatment against schistosomiasis) free of charge.

### 2.2. Study Area and Population

**Figure 1:**
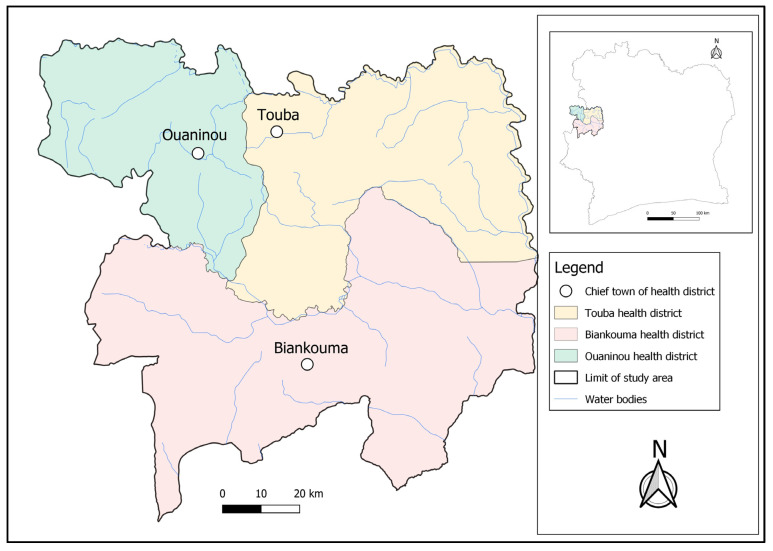
Map of the study area showing the health districts of Touba, Biankouma, and Ouaninou in the western part of Côte d’Ivoire, where three different sampling approaches for schistosomiasis were employed in August and September 2022.

The study was conducted in three health districts located in the western part of Côte d’Ivoire; namely, Touba (geographical coordinates: 8°40′0″ N latitude; 7°30′0″ W longitude) and Ouaninou (8°14′00″ N; 7°52′0″ W) in the Bafing region and Biankouma (7°45′0″ N; 7°40′0″ W) in the Tonkpi region (Figure 1). According to the 2021 national census, there were 262,850 and 1,387,909 inhabitants in the Bafing and Tonkpi regions, respectively. The climate is humid tropical, with two seasons: the rainy season that lasts from March to October, and the dry season from November to February. Annual precipitation ranges from 1200 to 2000 mm.

Coffee and cocoa are the main cash crops that provide an important source of income for the population in this part of Côte d’Ivoire [20]. Cashew nut cultivation is becoming an increasingly important aspect of the economy, particularly in the Bafing region. People in rural areas practice mainly subsistence agriculture, with rice, cassava, maize, bananas, and yams as staple foods [20]. For water supply and other domestic activities, people use rainwater, rivers, traditional wells, streams, fountains, small multi-purpose dams, ponds, tap water, and spring water [21]. Some domestic (e.g., washing dishes and clothes), economic (e.g., fishing), and recreational activities of communities (e.g., bathing and swimming) are associated with human–water contacts that govern schistosomiasis transmission [22]. Further details of the study area have been described elsewhere [23]. In 2014, Touba and Ouaninou health districts were both classified as hyper-endemic for schistosomiasis, while the health district of Biankouma was classified as moderately endemic. As a result, the Touba and Ouaninou districts received biannual treatment, while the Biankouma district received annual treatment until 2022 [24]. School-aged children (5–14 years) were the target population for this study.

### 2.3. Study Design and Inclusion Criteria

**Figure 2:**
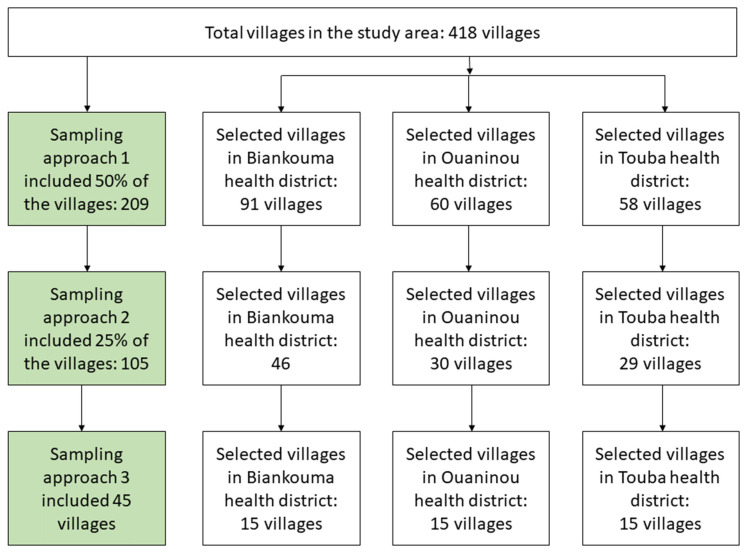
Study flowchart showing the different sampling approaches in the health districts of Biankouma, Ouaninou, and Touba in the western part of Côte d’Ivoire, in August and September 2022.

This study was a community-based, cross-sectional survey. It was conducted in August and September 2022 using three sampling approaches. The first approach included a random selection of 50% of the villages in each district. The second approach included a random selection of half of the approach 1 villages, hence, constituting 25% of the villages in each district. The third approach was the standard national procedure, including 15 villages that were randomly selected from approach 2 villages in each health district (Figure 2). Of note, the third approach was applied during the first national schistosomiasis mapping in 2013 and 2014 in Côte d’Ivoire [16] and served as the reference standard in the country.

The health districts provided lists of villages. After randomly selecting study villages, all three approaches followed the same procedures from the selection of households and participants. Compact segment sampling was employed to select participants within each selected village. This involved dividing the selected locality into segments of approximately 50 households using, whenever possible, existing boundaries such as rivers and roads, and then randomly selecting a single segment. All households in the selected segment were numbered, and about half of them were randomly selected per village based on an A or B pre-determined list of numbers. All school-aged children (5–14 years) from each selected household were invited to participate in the study. In each village, regardless of the approach, the participating children were the same individuals.

### 2.4. Parasitological Survey

After obtaining permission from village authorities, the day before stool sample collection, selected households were visited by a researcher with the support of community health workers (CHWs). The objectives and procedures of the study, including potential risks and benefits, were explained and written informed consent was obtained from parents or legal guardians. Children aged 9 years and above additionally provided assent. Each child was given a plastic container (125 mL) and invited to provide a fresh stool sample the following morning. The children’s demographic information, such as age and sex, was recorded. The filled containers were collected and labelled with unique identifiers, and transferred to nearby laboratories in the respective health districts for diagnostic work up. Eggs of *S. mansoni* were detected using the Kato–Katz thick smear method [25]. Each stool sample was subjected to duplicate Kato–Katz thick smears (slides A and B), using a 41.7 mg standard template. After a clearing time of 30–45-min, experienced laboratory technicians examined the slides under a microscope and recorded the number of *S. mansoni* eggs on summary sheets before entering them into tablets. For quality control, 10% of the slides were randomly selected and re-examined on the same day by a senior technician. In case of conflicting results, the slides were re-examined a third time and the results were discussed until agreement was reached [26].

### 2.5. Statistical Analysis

Data were entered directly in the field into tablets using open data kit (ODK) collect application (version 2022.3.6). The database was uploaded to a central server and extracted into an Excel format. Children were classified into two age groups (i.e., 5–8 years and 9–14 years). Data were analysed using R version 4.3.2 and STATA version 14.2 (StataCorp LLC; College Station, Texas, USA). The intensity of *S. mansoni* infection was determined by multiplying the sum of the two Kato–Katz thick smear readings with a factor of 12 and expressed as eggs per gram of stool (EPG). The intensity was stratified into three categories: light (1–99 EPG), moderate (100–399 EPG), and heavy (≥400 EPG). The prevalence was classified into three risk categories: low (<10%), moderate (10–49.9%), and high (≥50%) [7].

Generalized estimating equation models for binary outcomes with logit link functions and independent correlation structure were applied to estimate the prevalence and corresponding 95% confidence intervals (CIs). All models used robust variance estimators to account for potential correlation within clusters (i.e., village). The CIs were used to compare the different sampling approaches. The CIs for the geometric mean were not adjusted for clustering. Hence, the actual CIs might be slightly broader than the reported values. QGS version 3.24.1 was used to generate the map of the study area.

## 3. Results

### 3.1. Population Characteristics

Table 1 summarizes the demographic characteristics of the study population. The study included 209, 105, and 45 localities with 4964, 2441, and 1034 school-aged children for the first, second, and third sampling approach, respectively. The proportion of girls (47.6%, 46.6%, and 46.2%) and the mean age of the children (8.4 years, standard deviation (SD) 2.7 years; 8.4 years, SD 2.7 years; and 8.3 years, SD 2.6 years) were similar in the three approaches. The age groups presented similar proportions regardless of the sampling approach. The same patterns were observed within the health districts.

### 3.2. Prevalence of S. mansoni Infection

**Figure 3:**
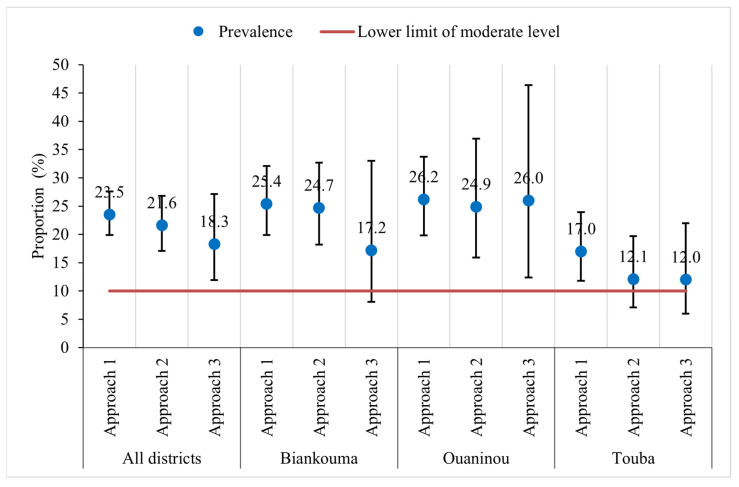
Prevalence of *S. mansoni* infection according to three sampling approaches in three health districts in the western part of Côte d’Ivoire, in August and September 2022.

The overall prevalence of *S. mansoni* was moderate in the study area: 23.5% (95% CI: 19.9–27.6%), 21.6% (95% CI: 17.1–26.8%), and 18.3% (95% CI: 11.9–27.1%) with the first, second, and third approach, respectively (Figure 3). At the unit of the health district, the estimated prevalence in Biankouma was 25.4% (95% CI: 19.9–32.1%), 24.7% (95% CI: 18.2–32.7%), and 17.2% (95% CI: 8.1–33.0%) for the first, second, and third approach, respectively. In Ouaninou, we found a prevalence of 26.2% (95% CI: 19.8–33.7%) for the first, 24.9% (95% CI: 15.9–36.9%) for the second, and 26.0% (95% CI: 12.4–46.4%) for the third approach. The prevalence in the Touba health district was estimated at 17.0% (95% CI: 11.8–23.9%), 12.1% (95% CI: 7.1–19.7%), and 12.0% (95% CI: 6.0–22.0%) for the three sampling approaches.

Based on the 95% CIs, we found similar prevalence estimates for the three sampling approaches. Similarly, at the unit of the health district, we observed that the estimated prevalence did not differ depending on the sampling approach. However, in the three health districts, the CIs are wider with the third approach compared to the other two. The prevalence obtained with the third approach is therefore less precise. The gaps are wider when comparing the third with the other two approaches in the districts of Ouaninou and Biankouma, while in Touba, they are quite small (Figure 3).

Table 2 shows the heterogeneity in the distribution of *S. mansoni* infection at the village level within the health districts, according to the third approach that focused on 15 randomly selected villages in each district. We observed a lower dispersion of prevalence in Touba (SD 15.3%) compared with Biankouma (SD 22.2%) and Ouaninou (SD 29.3%). These findings indicate a lower degree of heterogeneity in *S. mansoni* prevalence estimates in the Touba health district compared to the Biankouma and Ouaninou health districts.

**Figure 4:**
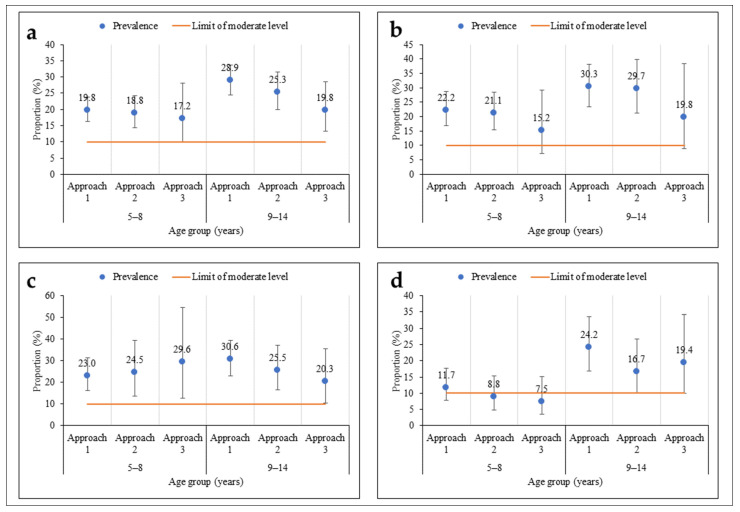
Prevalence of *S. mansoni* infection according to three sampling approaches stratified by participants’ age (5–8 and 9–14 years) in three health districts in the western part of Côte d’Ivoire, in August and September 2022 ((**a**) = all health districts; (**b**) = Biankouma; (**c**) = Ouaninou, and (**d**) = Touba).

As regards age, there was no significant difference in prevalence according to the sampling approach (Figure 4). The prevalences were moderate among children aged 5–8 years and their older counterparts (9–14 years), regardless of the sampling approach. However, among children aged 5–8 years, the prevalences were less precise using the third approach compared to the other two, more extensive sampling approaches. The same pattern was observed within the health districts, except in Touba, where the prevalence was low in the second and third approaches among children aged 5–8 years.

### 3.3. Intensity of S. mansoni Infection

**Figure 5:**
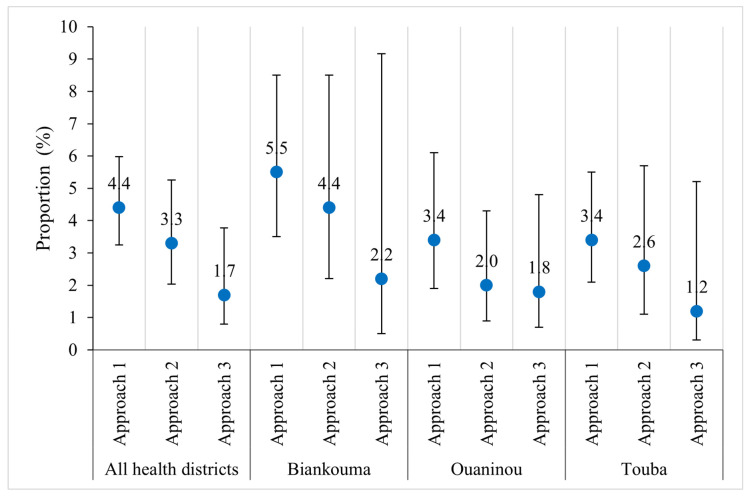
Proportions of heavy *S. mansoni* infections (i.e., ≥400 eggs per gram of stool, EPG) out of the total enrolled children, according to three sampling approaches in three health districts in the western part of Côte d’Ivoire in August and September 2022.

Moderate average infection intensities of *S. mansoni* were observed for the first two sampling approaches with an overall geometric mean of 117.9 EPG (95% CI: 109.3–127.3 EPG) in the first and 104.6 EPG (95% CI: 93.8–116.6 EPG) in the second approach. The third approach revealed a considerably lower mean intensity of 94.6 EPG (95% CI 79.5–112.7 EPG). In the three health districts, the infection intensities were generally moderate using any of the three approaches (Table 3). Except for the Touba health district, where the second approach exhibited the lowest precision, the precision of infection intensity decreases as the number of villages included in the approaches decreases. The same pattern was observed in the proportion of heavy intensity infections in the Biankouma health district. This is less evident overall and in the health districts of Ouaninou and Touba (Figure 5).

## 4. Discussion

In the new WHO roadmap, schistosomiasis is targeted for elimination as a public health problem by 2030 [27]. To achieve this ambitious goal, national control programmes implement preventive chemotherapy using praziquantel as the mainstay [28]. The current study evaluated the effectiveness of three sampling approaches, based on prevalence and intensity estimates of *S. mansoni* infection in school-aged children in three health districts in the western part of Côte d’Ivoire. The overall prevalence of *S. mansoni* in the three health districts was moderate (i.e., Biankouma, 17.2–25.4%; Ouaninou, 24.9–26.2%; and Touba, 12.0–17.0%). Our results corroborate previous findings from cross-sectional surveys pertaining to *S. mansoni* infection among school-aged children in the western part of Côte d’Ivoire [14,15].

The three sampling approaches revealed prevalence estimates of *S. mansoni* in the range of 12.0% to 26.2%. Hence, according to WHO guidelines [29,30], the three health districts require the same preventive chemotherapy strategy; namely, annual administration of praziquantel to all age groups from 2 years old and above, including adults, pregnant women after the first trimester, and lactating women [11,31]. However, based on the 95% CIs, we found that the prevalence according to the current standard approach (mimicking the third approach in our study) might fall below 10% in the health districts of Biankouma and Touba. The stratification of children into two age groups revealed that in the younger counterparts (aged 5–8 years) the overall prevalence was below 10%. These scenarios mean that preventive chemotherapy might only be necessary at the beginning of schooling and before leaving school.

Interestingly, comparing the overall prevalence and intensity of *S. mansoni* infection in the three sampling approaches, we observed a decrease in the prevalence and intensity as the number of surveyed villages decreased. This observation might be explained by the focal distribution of schistosomiasis [19], which seems to affect the overall prevalence and intensity according to the sampling approach. However, there was no difference between the three approaches regarding the overall endemicity category that guides the preventive chemotherapy intervention. According to the prevalence estimates in this study, approaches 1, 2, and 3 all lead the programme to the same decision regarding preventive chemotherapy in the three districts, namely annual treatment. It is important to note that mapping according to approach 3 is less resource intense and considerably cheaper. In the Biankouma health district, the first and second approaches revealed similar estimates of *S. mansoni* prevalence and intensity, whilst in the health district of Touba, the second and third approaches revealed similar estimates. It should be noted that in the health district of Biankouma, there were 91, 46, and 15 villages in the first, second, and third approach, respectively, while in the health district of Touba, there were 58, 29, and 15 villages surveyed. The low precision observed in the *S. mansoni* prevalence within health districts, particularly in Biankouma and Ouaninou, indicates that in areas with highly heterogeneous spatial distributions, using the current standard approach might misclassify the overall endemicity levels. In turn, this misclassification might result in a suboptimal frequency of preventive chemotherapy [18,32] and increase the risk of either under- or over-treatment [33]. Conversely, in health districts with less heterogeneous distribution—like the Touba health district in the current study—the number of villages included per district in the mapping process might have a negligible impact on the classification of endemicity levels. However, in very low-endemic areas, a small number of villages could have a considerable influence on the classification. Taken together, the choice of approach in mapping prevalence should consider the spatial distribution characteristics of the area to ensure precise classification and effective implementation of preventive chemotherapy and other interventions.

Given our findings, the first approach with sampling half of the villages in a health district provides more precise estimates of the prevalence and intensity of *S. mansoni* infection than the other less exhaustive sampling schemes. However, the second approach with 25% of villages included in the sampling might serve as a good compromise. Such an approach is less financially and human resource intensive and produces reasonably precise *S. mansoni* prevalence and intensity estimates. As our study was conducted in only three health districts in the western part of Côte d’Ivoire, care is indicated in the generalization of the results. To gain a deeper understanding of the implications of different sampling approaches, we need to extend this study to other health districts and evaluate resource requirements to also assess their cost-effectiveness.

## 5. Conclusions

The current study revealed that schistosomiasis remains a public health problem in the western part of Côte d’Ivoire. Regardless of the sampling approach used, the study area and the health districts of Biankouma, Ouaninou, and Touba were classified as moderately endemic for *S. mansoni* according to current WHO guidelines. Sampling approaches with a higher number of villages than the currently proposed 15-village sampling per health district provided a more precise estimate of the prevalence and intensity of *S. mansoni* infection. In health districts characterized by heterogeneous spatial distribution, the first and second approaches showed higher precision, and hence, more precise classification of the endemicity level. Conversely, in areas where there is little heterogeneity of the spatial distribution, the precision in prevalence was relatively consistent. As a pragmatic way forward, we recommend the current standard approach with only 15 villages per health districts be performed at a national scale, supplemented by more extensive sampling of at least 25% of villages in districts where there is high spatial heterogeneity and the prevalence is close to thresholds in WHO guidelines. Extending this study to other health districts of Côte d’Ivoire and other countries in sub-Saharan Africa is warranted to support or refute our conclusions. Future studies should include costing data to assess the cost-effectiveness of different sampling approaches for schistosomiasis control and elimination as a public health problem.

## Figures and Tables

**Table 1:** Demographic characteristics of the study population in the health districts of Biankouma, Ouaninou, and Touba in the western part of Côte d’Ivoire, in August and September 2022.

Characteristics	All Districts			Biankouma			Ouaninou			Touba		
	Approach 1	Approach 2	Approach 3	Approach 1	Approach 2	Approach 3	Approach 1	Approach 2	Approach 3	Approach 1	Approach 2	Approach 3
Overall	4964	2441	1034	2357	1161	360	1342	658	331	1265	622	343
**Sex**												
Female (%)	2363 (47.6)	1137 (46.6)	478 (46.2)	1152 (48.9)	548 (47.2)	171 (47.5)	623 (46.4)	299 (45.4)	137 (41.4)	588 (46.5)	290 (46.6)	170 (49.6)
Male (%)	2601 (52.4)	1304 (53.4)	556 (53.8)	1205 (51.1)	613 (58.8)	189 (52.5)	719 (53.6)	359 (54.6)	194 (58.6)	677 (53.5)	332 (53.4)	173 (50.4)
**Age group**												
5–8 years (%)	2913 (58.7)	1410 (57.8)	615 (59.5)	1388 (58.9)	673 (58.0)	198 (55.0)	784 (58.4)	372 (56.5)	203 (61.3)	741 (58.6)	365 (58.7)	214 (62.4)
9–14 years (%)	2051 (41.3)	1031 (42.2)	419 (40.5)	969 (41.1)	488 (42.0)	162 (45.0)	558 (41.6)	286 (43.5)	128 (38.7)	524 (41.4)	257 (41.3)	129 (37.6)
**Mean (SD) age** (in years)	8.4 (2.7)	8.4 (2.7)	8.3 (2.6)	8.3 (2.6)	8.4 (2.7)	8.5 (2.7)	8.4 (2.7)	8.5 (2.7)	8.3 (2.6)	8.4 (2.7)	8.4 (2.7)	8.1 (2.6)
**No. of villages**	209	105	45	91	46	15	60	30	15	58	29	15

% = percentage; No. = number.

**Table 2:** Prevalence of *S. mansoni* infection in villages of three health districts from the western part of Côte d’Ivoire, in August and September 2022, according to the third sampling approach with a random sample of 15 villages surveyed.

Health District	Study Village															SD
	1	2	3	4	5	6	7	8	9	10	11	12	13	14	15	
Biankouma	0.0	0.0	0.0	0.0	0.0	6.9	9.1	11.1	14.3	17.4	20.8	21.4	38.5	52.6	75.9	22.2
Ouaninou	0.0	0.0	4.0	4.4	5.3	6.1	7.7	9.1	10.5	11.1	27.3	37.5	57.1	82.4	86.1	29.3
Touba	0.0	0.0	0.0	3.0	3.2	3.7	4.4	6.3	6.7	10.5	13.6	18.8	18.9	36.8	54.2	15.3

SD = standard deviation.

**Table 3:** Infection intensity of *S. mansoni* by approach in three health districts in the western part of Côte d’Ivoire in August and September 2022.

	Approach 1 (GM, 95% CI)	Approach 2 (GM, 95% CI)	Approach 3 (GM, 95% CI)
All health districts	117.9 (109.3–127.3)	104.6 (93.8–116.6)	94.6 (79.5–112.7)
Biankouma	132.1 (118.7–146.9)	111.6 (95.6–130.2)	99.6 (70.9–140.0)
Ouaninou	100.9 (88.4–115.2)	93.0 (78.4–110.3)	104.6 (82.5–132.6)
Touba	110.9 (91.6–134.3)	105.5 (77.2–144.1)	71.0 (48.2–104.5)

CI = confidence interval; GM = geometric mean infection intensity, as expressed in eggs per gram of stool (EPG).

## Data Availability

All datasets used and/or analysed can be made available by the authors upon reasonable request.
